# What Matters in Online Education: Exploring the Impacts of Instructional Interactions on Learning Outcomes

**DOI:** 10.3389/fpsyg.2021.792464

**Published:** 2022-01-14

**Authors:** Xing Li, Xinyue Lin, Fan Zhang, Yuan Tian

**Affiliations:** ^1^Key Laboratory of Adolescent Cyberpsychology and Behavior (CCNU), Ministry of Education, School of Psychology, Central China Normal University, Wuhan, China; ^2^School of Psychology, Central China Normal University, Wuhan, China; ^3^School of Business and Management, Shanghai International Studies University, Shanghai, China

**Keywords:** instructional interactions, learning outcomes, task value, self-regulated learning, Interactive Equivalence Theory

## Abstract

Instructional interactions, which includes student–student interaction (SS), student–teacher interaction (ST), and student–content interaction (SC), are crucial factors affecting the learning outcomes in online education. The current study aims to explore the effects of instructional interactions on individuals’ learning outcomes (i.e., academic performance and learning satisfaction) based on the Interactive Equivalence Theory by conducting two empirical studies. In Study 1, we explored the direct relationships between instructional interactions and learning outcomes. A quasi-experimental design was used to manipulate the two groups of subjects (*n*_1_ = 192; *n*_2_ = 195), and the results show that not all of the three types of interaction can significantly positively predict learning satisfaction, among which ST cannot significantly predict learning satisfaction. When the total amount of instructional interactions is constant, adjusting the relative level of the three types of instructional interactions can effectively improve the learning outcomes to some extent. We further probed into the mediating effects of task value and self-regulated learning on the relationships between instructional interactions and learning outcomes in Study 2. We conducted an online survey and collected 374 valid data. The results showed that task values mediated the relationship between SS and learning satisfaction. In addition, SC can not only directly affect learning satisfaction, but also affect it through task value and self-regulated learning respectively, or *via* chain mediations of both task value and self-regulated learning. Our findings enrich the previous instructional interactions research and provide reference for online education curriculum design.

## Introduction

The number of online learning users has exploded during the COVID-19 pandemic (Nikou and Maslov, 2021), with face-to-face instruction being replaced by online instruction (Dong et al., 2020; Velle et al., 2020). According to the statistics of China Internet Network Information Center (CNNIC), by June 2020, the number of online education subscribers in China had reached 381 million, accounting for 40.5% of the total number of Internet users (China Internet Network Information Center (CNNIC), 2020). Online learning, also known as e-learning (Ray et al., 2021) and distance education (Devkota, 2021), is a kind of education pattern which exploits internet and information technology. In this educational pattern, mutual interactions, timely communication, independent learning and resource optimization are the exceeding vital teaching organization activities (Xie and Zhang, 2004). Instructional interactions are processes of interaction and communication between students and learning environment in the teaching process that helps achieve learning objectives (Wang, 2016). Instructional interactions are the primary factor influencing learning outcomes in online education (Mary and Xiao, 2014), and are crucial to improving learning support services (Yang and Mo, 2010).

Although a body of previous studies focused on the direct relationships between instructional interactions and learning outcomes, few existing studies have paid attention to the allocation of classroom instructional interactions resources, and the internal mechanism of the above direct relationships. In fact, online resources are so limited that it’s impossible to increase the variety of instruction indefinitely, especially during the pandemic (Mbydzenyuy and Silungwe, 2020). Issues about maximizing learning outcomes with limited resources need to be considered. Moreover, there exists inconsistent research conclusions about the direct relationships between instructional interactions and learning outcomes (Thurmond and Wambach, 2004; Bray et al., 2008; Sher, 2009; Kuo et al., 2013). Few researchers have explained this phenomenon. Even though it is speculated that there may be cognitive mechanisms involved in this direct relationship (Thorpe and Godwin, 2006), it has not been tested empirically. Therefore, this study intends to explore the optimization design of instructional interactions in online learning environment and the internal mechanisms of the relationships between instructional interactions and learning outcomes.

## Literature Review

### Instructional Interactions

The classical categories of instructional interactions contain student–student interaction (i.e., SS), student–teacher interaction (i.e., ST) and student–content interaction (i.e., SC) (Moore, 1989; Wang, 2016). SS refers to the process in which learners exchange knowledge, ideas or views on course content regardless of the presence of teachers; ST refers to the two-way communication between teachers and learners in the course of learning; SC refers to the process in which learners themselves explain and reflect on the learning topic or content. SS and ST are collectively known as interpersonal interaction (Thorpe and Godwin, 2006). Moore holds that all three types of instructional interactions are indispensable to ensure the success of online education, and the overall level of instructional interactions need to be constantly improved to ensure the maximum level of each type of instructional interactions (Bernard et al., 2009).

Anderson (2003) holds different views on the configuration of the types and quantities of instructional interactions in the course. They found that there are equivalent substitutions among the three types of instructional interactions in the process of affecting learning outcomes. The Interactive Equivalence Theory are therefore put forward. The core ideas of the theory are as follows: (1) As long as the level of one of instructional interactions is very high, it is enough to produce profound and meaningful learning, while the other two could be offered minimally, or even eliminated without degrading the teaching experience; (2) Even if the overall level of instructional interaction is not high and the amount of time and energy spent is not much, when more than one type of instructional interactions is high, it is likely to bring a more satisfying educational experience than those online education that takes more time and energy. That is, instructors can design only one or two kinds of instructional interactions by analyzing the needs of students in a specific learning environment, which can reduce teaching cost and improve flexibility on the premise of hardly losing teaching efficiency.

Likewise, Bernard et al. (2009) discovered that although higher and middle levels improved learning outcomes more than lower levels when considering the total level of instructional interaction, it did not mean that increasing any one kind of instructional interaction can improve learning outcomes. Instead, only increasing SC can the effect value of instructional interactions on learning outcomes be significantly increased. Some researchers also affirmed that SS or ST is not a necessary part of teaching. Instructional designs combined with “SS and SC” or “ST and SC” are better than the one combined with three instructional interactions (Bernard et al., 2009; Miyazoe and Anderson, 2012).

If equivalent substitution exists, appropriate instructional design can make a small number of instructional interactions achieve better learning outcomes. However, to date, a vast number of researches focus on how to improve the number of instructional interactions, ignoring the limited resources of learning resources. Therefore, it is necessary to pay attention to the maximize utilization of limited resources. We thus hypothesized: (H1) When the total amount of instructional interaction is constant, alterations in the relative level of SS, ST, and SC will lead to significantly different learning outcomes.

### Learning Outcomes

The measurement indexes of online learning outcomes mainly include academic performance, learning satisfaction, continuous learning behavior or willingness (Ding and Wu, 2005). Previous studies have always regarded academic achievement as the representative of learning outcome in general (Joksimovic et al., 2015; Sucipto et al., 2017; Nabizadeh et al., 2019; Amer, 2020). Academic performance is an objective quantitative indicator of learning outcomes (Krchner et al., 2021), which represents the achievement of teaching objectives and students’ mastery of knowledge. It is worth noting that academic performance should consider not only students’ examination performance but also their usual performance, such as learning enthusiasm, creativity and teamwork ability (Zhang, 2011). For example, Beldarrain (2008) defined the academic performance of online learning as the accumulated performance after learning at least 50% of the content of a course, including two module exams and additional course activities.

Unlike the popular use of academic performance in evaluating individuals’ learning outcomes, affective variables are overlooked. Learning satisfaction is a quantitative indicator of students’ overall satisfaction with online learning needs (Yang et al., 2014). Wan et al. (2017) proposed that user satisfaction should be taken as the key points of distance education resource administration given that it is key reference standard for online education service quality and learning outcomes. Understanding students’ learning satisfaction is conducive to improving online education service quality and improving teaching quality evaluation system (Zeng et al., 2016; Jiang et al., 2021). A large number of studies have consistently agreed that learning satisfaction can positively and significantly predict continuous learning behavior or willingness (Angelova and Zekiri, 2011; Faize and Nawaz, 2020). It is representative to measure the affective cognition index of online learning by learning satisfaction (Faize and Nawaz, 2020; Jiang et al., 2021). Therefore, this study selects indicators of learning satisfaction and academic performance to measure learning outcomes subjectively and objectively.

### The Impacts of Instructional Interactions on Learning Outcomes

The principle of interactive determinism in the Social Learning Theory conceives that the study of learning should not ignore the influence of social variables on human behaviors, thus emphasizing the role of observational learning, indirect experience and role models (Bandura, 1978). The indirect experience acquired by the learners through observing the behaviors of their peers or teachers in the learning process can play a role of substitute reinforcement for the learners, and thus facilitating their acquisition of corresponding behaviors.

The process of interpersonal interaction during online learning allows learners to have more opportunities to observe and learn, obtain indirect experience and find learning models, which has a certain impact on the satisfaction of students’ learning expectations. If the degree of interpersonal interaction (i.e., SS and ST) does not meet expectations, students will be hindered in their learning process, feeling isolated psychologically, weakening their interest in learning, and thus causing dissatisfaction (Kuo et al., 2013, 2014). In addition, the online education’s “separation” of time and space characteristics make direct experience for big discounts. Students are more likely to observe and learn by browsing the content on the platform, which leading to the learning quality depending largely on the interaction level between students and the platform content. The effective interaction content design can reduce network losses, improve learning satisfaction and academic performance (Xiao, 2017). Therefore, we propose that: (H2) Instructional interactions (SS, ST, and SC) can significantly positively predict learning outcomes.

### The Mediating Role of Task Value

The motivation of an individual to complete a certain task is determined by his/her expectation of the possibility of success of the task and the subjective value given to the task (Eccles and Harold, 1991). In online courses, the more likely students are to achieve their goals and the more value they will gain from the course, the more motivated they will be to complete the course tasks. Task value refers to students’ perception of the importance, interestingness, meaning of helping others, practical value and cost of course tasks (Eccles and Harold, 1991; Qi and Fang, 2005; Gong et al., 2016).

Task value plays a key role in the success of learning outcomes. When the task value of online courses is assessed as high level by students, they will tend to give higher overall evaluation and have more satisfactory experience (Lei et al., 2017). In other words, learning satisfaction will increase when individuals perceive the value certain things brings to them to meet expectations (Johnson et al., 2014). Furthermore, task value is determined by the characteristics of the course task itself and the needs of the learners themselves (Qi and Fang, 2005). Given task characteristics and individual needs will change correspondingly due to the intervention of external environment, task value perceived by individuals is not invariable. A tracking study shows that students’ task value and self-efficacy can be changed with external effects (Johnson et al., 2014). Instructional interactions in online education are external stimulus provided to learners. When these stimuli act on individuals, they can arouse feelings of course value perception, such as interest and rewards. Through assimilation and compliance, this value perception can promote the creation of a high level of learning satisfaction experience (Sun and Li, 2011). In short, this study proposes that: (H3) Learners’ task value perception can play a mediating role between instructional interactions and learning outcomes.

### The Mediating Role of Self-Regulated Learning

Initial proposed in the Social Learning Theory (Bandura, 1978), self-regulated learning is further defined by Zimmerman (1989) as the process in which learners actively manage, promote and participate in their own learning activities in terms of metacognition, motivation and behavior. Based on the interactive determinism principle in the Social Learning Theory (Bandura, 1978), students’ self-regulated learning will be affected by social and physical environment (Sun and Li, 2011).

Instructional interactions can promote information exchange between learners and teaching elements, such as teachers, classmates and learning resources, which guide learners to constantly adjust their learning according to their own cognitive structure. In the online education environment, the communication and cooperation between students, classmates and teachers are conducive to solve curriculum problems, stimulate students’ learning motivation, and promote the autonomy of learning. The interaction between students and contents gives students autonomy in mastering learning progress. If students can control and adjust their learning autonomously in a planned way during the learning process, the possibility of success in online learning will be greater (Dai, 2013), thus improving their learning satisfaction. Some researchers have confirmed that students’ self-regulated learning plays a mediating role in the relationship between academic orientation and learning outcomes (Duan and Zhang, 2010). Therefore, this study proposes that (H4): self-regulated learning may play a mediating role between instructional interactions and learning outcomes.

In addition, since the more value students gain from the course, the more motivated they will be to complete the course tasks (Eccles and Harold, 1991), high-value tasks can make individuals get more rewards after completing the course, which stimulates students’ learning motivation, and induces students’ autonomous learning behavior (Qi and Fang, 2005). The higher the perceived task value of students in online courses, the more likely they are to show more self-regulated learning (Freeze et al., 2010). Therefore, this study further proposes that (H5): instructional interactions may also affect learning outcomes through the chain mediation of both task value and self-regulated learning.

### The Present Study

To test our theoretical hypotheses, we are aimed to conduct two empirical studies. In Study 1, we adopt a quasi-experimental study to examine the direct relationships between instructional interactions and learning outcomes. Once the direct relationships are confirmed in Study 1, we would further explore the internal mechanisms underlying the direct relationships by conducting an online survey In Study 2.

**FIGURE 1:**
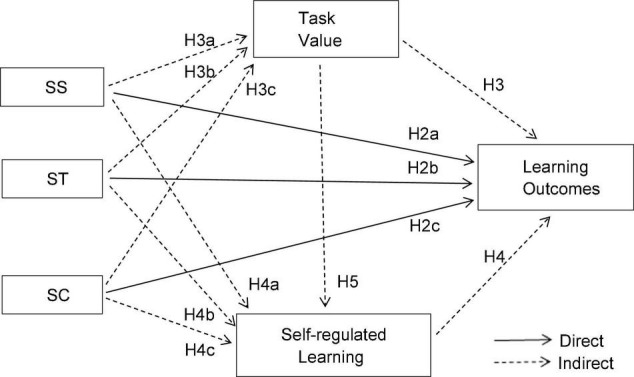
Conceptual model. SS, student–student interaction; ST, student–teacher interaction; SC, student–content interaction. Similarly hereinafter.

Specifically, we expect the following relationships (H2–H5 can be seen in Figure 1): Learning outcomes can be effectively improved by adjusting the relative level of SS, ST, and SC as required (H1); and three types of instructional interaction can positively predict learning outcomes (H2a–H2c). In addition, instructional interaction can affect learning outcomes through the simple mediating effect of task value (H3a–H3c), self-regulated learning (H4a–H4c) and the chain mediating effect of “task value → self-regulated learning” (H5a–H5c).

## Study 1

A quasi-experimental study was carried out to examine the direct relationships between instructional interactions and learning outcomes. We manipulated instructional interactions (i.e., total instructional interaction, SS, ST, SC) between two online classrooms by referring to the Interaction Equivalence Theory. Academic performance and learning satisfaction were devoted to measure the learning outcomes. Then the differences of students’ learning outcomes under two instructional interaction designs were investigated, as well as the relationship between instructional interactions and learning outcomes.

### Method

#### Participants and Procedure

The students who chosen online course of General Psychology in a university in Hubei, China, were randomly divided into two classes (i.e., Class 1 and Class 2). We collected 401 subjects’ data in the two classes, and a total of 387 valid subjects were retained, with 192 from Class 1 and 195 from Class 2. The age distribution of the subjects ranged from 18 to 23 years old (*M* = 20.15, *SD* = ±1.02). In total, 86% of the subjects mastered the basic computer operation.

Participants were randomly divided into two classes, who shared the same course content and same tutors. The instructional interactions levels were manipulated by designing teaching. After the course was conducted for a period of time, we took questionnaires to measure the instructional interactions level and learning outcomes in different classes. The independent variables of this study were the level of three kinds of instructional interactions between two classes. Class 1 paid more attention to interpersonal interaction (SS and ST), such as participating in the discussion of questions raised by peers in the discussion forum, asking and answering teachers’ questions through emails and discussion forums, etc. Class 2 pays more attention to the interaction between students and the learning content (SC), for example, more students are required to read texts or watch videos in the course task description.

In addition, in order to ensure that the field teaching experiment complies with psychological ethics and on the premise of achieving the research purpose, the developmental differences brought by the experimental operation to different classes of subjects should be reduced as much as possible. When manipulating the number of different kinds of instructional interactions, the total number of instructional interactions in the two classes is as consistent as possible. Moreover, we make course schedule, curriculum content, time, teachers, exam content, and goals consistent. To avoid the impress of proactive inhibition, course selection was restricted in the course elective system, students in the two classes had neither attended the courses offered by the relevant teachers nor studies the general courses of psychology in advance in order to ensure the objectivity and fairness of the academic performance, the teacher and teaching assistant will mark the grades separately in the evaluation of ordinary grades and exam scores. If the difference between the scores of the two raters is more than 10 points, the scores will be invalid and the scores will be graded again. The average valid scores given by the two raters will be recorded as the students’ grades.

#### Measures

##### Instructional Interactions

**FIGURE 2:**
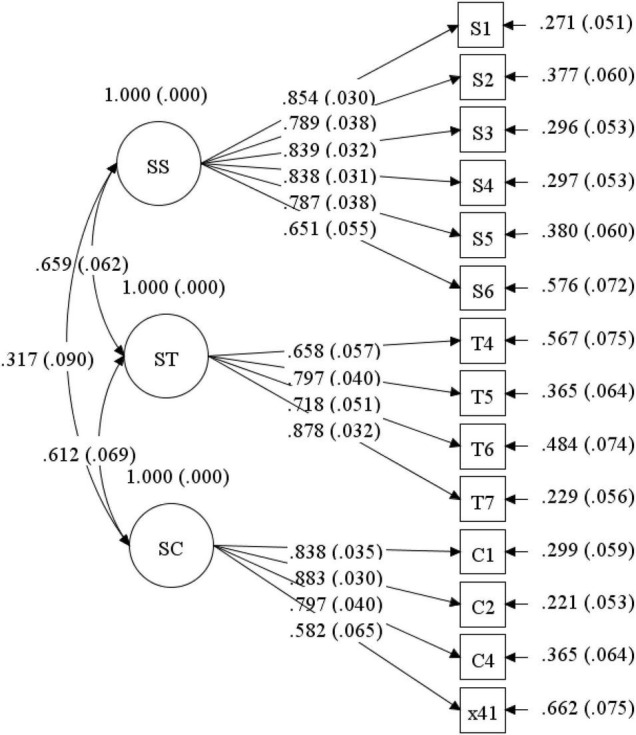
Confirmatory factor analysis (CFA) of instructional interactions.

This scale includes 19 items to measure three dimensions: SS (eight items), ST (seven items), and SC (four items) (Kuo et al., 2013, 2014). Each item was scored by five-points Likert-type (from 1 “very inconsistent” to 5 “very consistent”). The higher the score, the more interaction in learning. After item analysis and exploratory factor analysis, five questions with ambiguity, no significant difference between high group and low group, total correlation too large (>0.8) or too small (<0.3), and factor load less than 0.3 were deleted. Finally, a 14-item instructional interaction scale with three dimensions was obtained (see Table 1), which can explain 66.48% of variance. The scale has high reliability and validity: Cronbach’s α coefficient is 0.91; Confirmatory factor analysis showed that the three dimensions were well fitted (χ^2^/*df* = 2.29, RMSEA = 0.16, SRMR = 0.06, CFI = 0.92, TLI = 0.90) (see Figure 2).

**TABLE 1:** Factor loading of each item of instructional interactions.

Item	Factor loading		
	
	Factor 1	Factor 2	Factor 3
SS1	0.811		
SS3	0.806		
SS2	0.795		
SS5	0.789		
SS4	0.781		
SS6	0.557		
SC2		0.901	
SC3		0.835	
SC1		0.709	
SC4		0.462	
ST7			−0.803
ST4			−0.700
ST5			−0.697
ST6			−0.518

*SS, student–student interaction; ST, student–teacher interaction; SC, student–content interaction. Similarly hereinafter.*

##### Learning Satisfaction

The seven-item was adapted from the scales developed by Kuo et al. (2014) and Gong et al. (2016). Five-points Likert-type were used for each item (from 1 “very inconsistent” to 5 “very consistent”). The higher the score, the higher the learner’s satisfaction with online education. After conducting item analysis, two questions with a total correlation greater than 0.8 were deleted, thus resulting good reliability and validity. Factor loads of items ranged from 0.65 to 0.87, and the explanatory rate of measure variance was 68.20%. Confirmatory factor analysis showed that the three dimensions were well fitted (χ^2^/*df* = 2.20, RMSEA = 0.06, SRMR = 0.02, CFI = 0.99, TLI = 0.98). The Cronbach’s α coefficient of this questionnaire was 0.88.

##### Academic Performance

The index of academic achievement consists of usual performance and examination performance: usual performance throughout the course is based on the length of students’ online learning, online tests, frequency and quality of discussions, group collaboration and so on; Examination performance is mainly based on the recognition and reproduction of memorized knowledge in the test paper; Academic performance = 0.7 × usual performance + 0.3 × examination performance. In order to ensure the objectivity and fairness these scores, the lecturers and teaching assistants will mark them separately.

### Results

#### Preliminary Analysis

Harman single factor test (Zhou and Long, 2004) was carried out, and the results showed that there were four factors whose characteristic root was greater than 1, and the variation explained by the first common factor was 36.83% (<40%). The data could exclude the existence of serious common method deviation. Correlation analysis of all variables showed that variables were positively and significantly correlated with each other (see Table 2). Except that the correlation coefficient between total instructional interaction and SS is 0.83, the correlation coefficient between other variables is less than 0.80. And the variance inflation factor (VIF) showed that it is greater than and close to 1 (see Table 3), which can exclude the problem of multi-collinearity between variables (Field, 2000).

**TABLE 2:** Correlations of instructional interactions and learning satisfaction.

	SS + ST + SC	SS	ST	SC	Learning satisfaction
SS + ST + SC	1				
SS	0.83**	1			
ST	0.72**	0.32**	1		
SC	0.71**	0.30**	0.52**	1	
Learning satisfaction	0.67**	0.41**	0.55**	0.67**	1

N = 384, **p < 0.01, values are reserved for two decimal places, same as below.

**TABLE 3:** Regression analysis of instructional interactions on learning satisfaction.

Explained variable	Learning satisfaction			Learning satisfaction
	
Explaining variable	SS	ST	SC	Total instructional interaction
β	0.13	0.03	0.80	0.56
Standard error	0.05	0.07	0.08	0.04
*t*	2.43*	0.43	10.62***	13.48***
*p*	0.02	0.67	0.00	0.00
Tolerance	0.72	0.56	0.72	
VIF	1.38	1.78	1.39	
*R*	0.58			0.59
*R* ^2^	0.34			0.34
Adjusted *R*^2^	0.34			0.34
*F*	64.78***			181.67***
*p*	0.00			0.00

*p < 0.05, ***p < 0.001.

*For ethical reasons, only learning satisfaction was used to measure learning outcomes in regression analysis. Similarly hereinafter.*

#### Independent Sample *t*-Test

In order to explore the differences in the dimensions of instructional interactions and learning outcomes in the two classes, independent sample *t*-test was conducted for analysis. The results showed that (see Table 4) total instructional interaction (SS + ST + SC) (*t* = 1.46, *p* = 0.15) and examination performance (*t* = 1.67, *p* = 0.10) of the two classes were not significantly different. The SS (*t* = 2.41, *p* < 0.05) and ST (*t* = 2.53, *p* < 0.05) in Class 1 were significantly greater than those in Class 2. In addition, The SC (*t* = −2.51, *p* < 0.05), learning satisfaction (*t* = −2.24, *p* < 0.05), academic performance (*t* = −4.13, *p* < 0.001) and usual performance (*t* = −4.52, *p* < 0.001) in Class 1 were significantly lower than those in Class 2.

**TABLE 4:** Independent sample *t*-test.

	*M* ± *SD*		*t*
	
	Class 1 (*N* = 192)	Class 2 (*N* = 195)	
Total instructional interaction	3.99 ± 0.39	3.92 ± 0.46	1.46
SS	3.74 ± 0.56	3.59 ± 0.64	2.41*
ST	4.21 ± 0.46	4.08 ± 0.54	2.53*
SC	4.14 ± 0.45	4.27 ± 0.57	−2.51*
Learning satisfaction	4.08 ± 0.49	4.19 ± 0.52	−2.24*
Academic performance	82.95 ± 7.64	87.03 ± 13.29	−4.13***
Examination performance	88.924 ± 7.00	87.39 ± 12.38	1.67
Usual performance	80.38 ± 9.74	86.51 ± 18.63	−4.53***

*p < 0.05, ***p < 0.001.

*Total instructional interactions = SS + ST + SC.*

*Academic performance = 0.7 × usual performance + 0.3 × examination performance.*

*Learning satisfaction and academic achievement are two indicators of learning outcomes. Similarly hereinafter.*

#### Regression Analysis

In order to ensure the authenticity and validity of the data, all variables except academic achievement were measured anonymously. That is, academic achievement does not correspond to other variables in the study at individual level. Therefore, regression analysis between academic achievement and other variables was not possible. When it came to regression analysis, only learning satisfaction was used to measure learning outcomes.

The study only conducts regression analysis on learning satisfaction of, SS, ST, and SC. The results showed that (see Table 3) total Instructional Interaction (SS + ST + SC) (β = 0.56, *t* = 13.48, *p* < 0.001), SS (β = 0.13, *t* = 2.43, *p* < 0.05), SC (β = 0.80, *t* = 10.62, *p* < 0.001) can significantly positively predict learning satisfaction; ST (β = 0.03, *t* = 0.43, *p* = 0.67) had no significant effect on the prediction of satisfaction.

### Discussion

The results of *t*-test showed that there was no significant difference in total instructional interaction. The developmental difference between different groups of subjects brought about by experimental operation could be reduced by post-study intervention to some extent. Although the total instructional interaction between the two classes was almost the same, there were significant differences in SS, ST, and SC. That is, the degree of SS and ST in Class 1 was significantly higher than that in Class 2, and the degree of SC in Class 1 is significantly lower than that in Class 2. In addition, the learning outcomes (i.e., learning satisfaction and academic performance) of Class 1 was less than that of Class 2. Although there is no difference in total instructional interaction, the learning outcomes will be changed significantly due to differences in the level of three types of instructional interactions. These results verify H1.

The mean values of instructional interactions and learning satisfaction in the two classes was greater than 3, indicating that instructional interactions design did exist in the process of online education, and students were generally satisfied with online education. Moreover, there were significantly positive correlations between instructional interactions and learning satisfaction. Regression analysis results further showed that SS and SC has positive prediction function to the learning satisfaction, while ST cannot significantly predict learning satisfaction, which verifies the H2a and H2c.

In sum, not every type of instructional interaction can significantly predict learning satisfaction, and improving total instructional interaction didn’t necessarily improve learning outcomes. On the contrary, when total instructional interaction was fixed, adjusting the relative levels of SS, ST, and SC can effectively improve learning satisfaction. These results demonstrated the applicability of Anderson’s (2003) Interaction Equivalence Theory.

## Study 2

After confirming the direct relationship between instructional interactions (i.e., SS, ST, and SC) and learning satisfaction, we further obtained learner data from online education websites to explore the internal mechanism of instructional interactions affecting learning satisfaction.

### Method

#### Participants and Procedure

Participants were recruited through course announcements and email notifications on online education platforms. In total, 421 questionnaires were collected, and invalid answers were deleted in accordance with the principles of “three standard deviations” and “repeated questions continuously and regularly.” In order to ensure the directivity of the research results, the data of subjects with a very small proportion of college or below were excluded. Finally, 374 valid data were obtained, with an effective rate of 88.83%. There were 127 male and 247 female, with an average age of 21.90 (*SD* = 5.43) years. Undergraduates accounted for more than 90% of the total effective subjects. The majors of liberal arts (24.37%), science (23.11%), engineering (21.97%) and business (19.15%) accounted for more in the subjects. More than 85% of the subjects mastered basic computer operations.

#### Measures

##### Instructional Interactions

Same as the scale used in Study 1, The reliability and validity of this part are also great: Cronbach’s α coefficient was 0.91; The results of confirmatory factor analysis showed that the dimensions of the scale were well fitted (χ^2^/*df* = 2.29, RMSEA = 0.16, SRMR = 0.12, CFI = 0.68, TLI = 0.64).

##### Learning Satisfaction

Same as the scale used in Study 1, reliability and validity in this part are great: Cronbach’s α coefficient was 0.88; Confirmatory factor analysis showed that the fitting index of the scale was good (χ^2^/*df* = 2.20, RMSEA = 0.06, SRMR = 0.02, CFI = 0.99, TLI = 0.98).

##### Task Value

The scale which measures learners’ assessment and perception of the interest, significance and serviceability of the online course, is compiled by Artino (2008) and Gong et al. (2016). It consists of six questions scored by seven-point Likert-type scale (from 1 “very inconsistent” to 7 “very consistent”). We deleted one item with factor load less than 0.5 and obtained high reliability and validity: Cronbach’s α coefficient was 0.84; Confirmatory factor analysis showed that the three dimensions were well fitted (χ^2^/*df* = 2.96, RMSEA = 0.07, SRMR = 0.02, CFI = 0.99, TLI = 0.98).

##### Self-Regulated Learning

The scale developed by Zhu et al. (2005) contains two dimensions of motivation adjustment and strategy adjustment, with a total of 69 questions scored by six-point Likert-type scale (from 1 “very incompatible” to 6 “very compatible”). The higher the score, the stronger the learner’s self-regulated learning ability (Wang et al., 2010). The questionnaire had high reliability and validity: Cronbach’s α coefficient was 0.88; Confirmatory factor analysis showed that the two dimensions were well fitted (χ^2^/*df* = 3.01, RMSEA = 0.07, SRMR = 0.09, CFI = 0.68, TLI = 0.67).

### Results

#### Preliminary Analysis

Harman single factor test was performed on the variables in this study (Zhou and Long, 2004), and the results showed that there were 17 factors whose characteristic root was greater than 1, and the variation explained by the first common factor was 24.30% (<40%). The data in this study could exclude the existence of serious common method deviation.

Table 5 presented the mean, standard deviation and correlation results of the main variables. The average scores on every questionnaire were above the median. And the correlation analysis of variables shows that the variables are positively and significantly correlated with each other. In addition, VIF shows that it is greater than 1 and much less than 10, which can exclude the problem of multi-collinearity between variables (Field, 2000).

**TABLE 5:** Mean values, standard deviations and correlation coefficients.

Variables	*M* ± *SD*	SS	ST	SC	Task value	Self-regulated learning	Learning satisfaction
SS	3.35 ± 0.77	1					
ST	3.63 ± 0.69	0.55**	1				
SC	4.27 ± 0.53	0.29**	0.53**	1			
Task value	5.79 ± 0.77	0.29**	0.36**	0.57**	1		
Self-regulated learning	4.36 ± 0.46	0.29**	0.26**	0.32**	0.30**	1	
Learning satisfaction	4.33 ± 0.48	0.27**	0.50**	0.69**	0.60**	0.35**	1

**p < 0.01.

#### SEM Analysis

We employed Mplus 7.0 software to conduct structural equation modeling to test the paths of instructional interactions (i.e., SS, ST, SC), self-regulated learning, and task value affecting learning satisfaction by using maximum likelihood estimation (ML) method. The results showed that the fitting indexes of structural equation model were good (χ^2^/*df* = 4.37, RMSEA = 0.09, CFI = 0.94, TLI = 0.92, SRMR = 0.05) when the gender and computer proficiency of learners were controlled.

**FIGURE 3:**
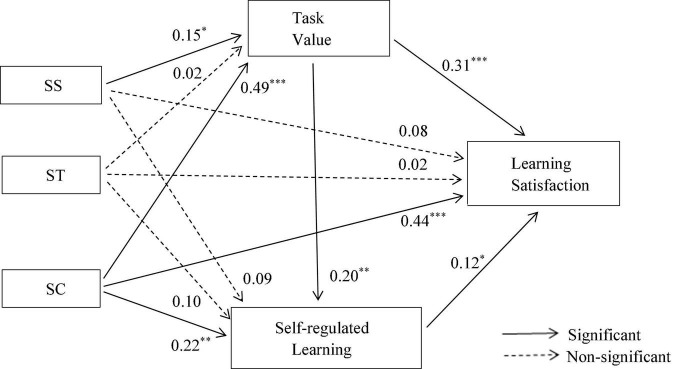
Influence path of instructional interactions on learning satisfaction. *p < 0.05, ^**^p < 0.01, ^***^p < 0.001. Control variables are not presented.

The path analysis results showed (see Figure 3 and Table 6) that the SS had significant effect on task value (β = 0.15, *Z* = 2.17, *p* < 0.05), while non-significant effect on self-regulated learning (β = 0.09, *Z* = 1.13, *p* > 0.05) and learning satisfaction (β = 0.08, *Z* = 1.50, *p* > 0.05); ST had non-significant effect on task value (β = 0.02, *Z* = 0.31, *p* > 0.05), self-regulated learning (β = 0.10, *Z* = 1.23, *p* > 0.05), and learning satisfaction (β = 0.02, *Z* = 0.41, *p* > 0.05); however, SC significantly impacted on task value (β = 0.49, *Z* = 9.19, *p* < 0.001), self-regulated learning (β = 0.22, *Z* = 3.18, *p* < 0.05), and learning satisfaction (β = 0.44, *Z* = 8.33, *p* < 0.001); Moreover, task value could significantly influence self-regulated learning (β = 0.20, *Z* = 3.16, *p* < 0.05) and learning satisfaction (β = 0.31, *Z* = 6.03, *p* < 0.001); Self-regulated learning also significantly affected learning satisfaction (β = 0.12, *Z* = 2.21, *p* < 0.05).

**TABLE 6:** Variable relation of regression analysis.

Regression equation		Fitting index		Regression coefficient and significance			
		
Explained variable	Explaining variable	*R*	*R* ^2^	β	*95% CIs*		*Z*
					
					Lower 2.5%	Upper 2.5%	
Learning satisfaction	Gender	0.71	0.50	0.04	–0.18	0.45	0.99
	Computer proficiency			0.03	–0.18	0.45	0.66
	SS			0.14*	0.02	0.12	2.39
	ST			0.04	–0.04	0.10	0.67
	SC			0.62***	0.86	1.10	13.70
Task value	Gender	0.58	0.34	0.01	–0.50	0.74	0.27
	Computer proficiency			0.12*	0.21	0.97	2.50
	SS			0.15*	0.02	0.16	2.17
	ST			0.02	–0.08	0.12	0.31
	SC			0.49***	0.74	1.06	9.19
Self-regulated learning	Gender	0.52	0.27	0.06	–0.69	3.43	1.10
	Computer proficiency			0.17**	1.22	3.91	3.09
	SS			0.09	–0.07	0.41	1.13
	ST			0.10	–0.08	0.56	1.23
	SC			0.22*	0.62	1.86	3.18
	Task value			0.20*	0.29	0.90	3.16
Learning satisfaction	Gender	0.76	0.58	0.03	–0.21	0.64	0.80
	Computer proficiency			–0.03	–0.40	0.15	–0.73
	SS			0.08	–0.01	0.09	1.50
	ST			0.02	–0.04	0.07	0.41
	SC			0.44***	0.55	0.82	8.33
	Task value			0.31***	0.19	0.34	6.03
	Self-regulated learning			0.12*	0.01	0.06	2.21

*p < 0.05, **p < 0.01, ***p < 0.001.

*Boot CI lower limit and Boot CI upper limit refer to the lower limit and upper limit of 95% confidence interval obtained by 5,000 times extraction of percentile Bootstrap method for deviation correction, the same as below.*

#### Mediation Analysis

We then still employed Mplus 7.0 software to conduct bootstrapping test (5,000 times) to respectively evaluating the mediation effects of task value and self-regulated learning between SS, ST, and SC and learning satisfaction. If zero is not between the upper and lower limits of 95% confidence interval, the indirect effect is significant (Hayes, 2009). The results of mediation analysis (see Table 7) showed that as for the indirect effects of SS on learning satisfaction, only the path of “SS → task value → learning satisfaction” was significant, with an effect value of 0.05 (95% CI = [0.01, 0.10] exclude 0), accounting for 35.71% of the total effect; The direct and indirect paths between ST and learning satisfaction were not significant; Among the indirect pathways of SC affecting learning satisfaction, the paths of “SC → self-regulated learning → learning satisfaction,” “SC → task value → learning satisfaction,” and “SC → self-regulated learning → task value → learning satisfaction,” were all significant, and the effect values were 0.03 (95% CI = [0.01, 0.10] exclude 0), 0.15 (95% CI = [0.01, 0.10] exclude 0) and 0.01 (95% CI = [0.01, 0.10] exclude 0), which accounted for 5.84%, 24.19%, and 1.61% of the total effect, respectively.

**TABLE 7:** Hypothetical path test.

Hypothesis	Path	Effect	*95% CIs*		Relative mediating effect (%)	Whether the hypothesis is validated
			
			Lower 2.5%	Upper 2.5%		
H2a	SS → learning satisfaction	0.08	−0.01	0.09		No
H3a	SS → task value → learning satisfaction	0.05	0.01	0.10	35.71	Yes
H4a	SS → self-regulated learning → learning satisfaction	0.01	−0.01	0.03		No
H5a	SS → task value → self-regulated learning → learning satisfaction	0.00	−0.01	0.01		No
H2b	ST → learning satisfaction	0.02	−0.04	1.10		No
H3b	ST → task value → learning satisfaction	0.01	−0.03	0.04		No
H4b	ST → self-regulated learning → learning satisfaction	0.01	−0.01	0.03		No
H5b	ST → task value → self-regulated learning → learning satisfaction	0.00	−0.01	0.00		No
H2c	SC → learning satisfaction	0.44	0.82	1.10		Yes
H3c	SC → task value → learning satisfaction	0.15	0.11	0.20	24.19	Yes
H4c	SC → self-regulated learning → learning satisfaction	0.03	0.01	0.05	5.84	Yes
H5c	SC → task value → self-regulated learning → learning satisfaction	0.01	0.01	0.02	1.61	Yes

### Discussion

The results of the direct relationship were consistent with Study 1, and complement the mediating variables. SS and SC can influence learning satisfaction through the mediating effect of task value. H3a and H3c are verified. This suggested that task value was a key factor influencing learning satisfaction in SS and SC. When students perceive that the course is useful and meaningful, they are more likely to be satisfied with their expectations, then, generate satisfaction experience.

Furthermore, SC affected learning satisfaction through the mediation of self-regulated learning, which verifies H4c. Online learning means that students will have fewer external constraints in learning, so it requires students’ self-regulation more than traditional learning (Artino, 2008). According to Moore (1989), SC refers to students’ self-elaboration and reflection on learning, regardless of whether others are present. More interaction with course content means more autonomy. Self-regulated learning is a process in which individuals manage their own learning activities. In the online learning environment, if students can effectively arrange and adjust their learning, they will be more likely to participate in the course learning and have a high degree of learning satisfaction (Xu et al., 2017).

In addition, SC can influence students’ learning satisfaction through the chain mediating effect of task value → self-regulated learning, which verifies H5c. From the perspective of motivational psychology, it can be seen that expectation and task value can improve students’ learning motivation, facilitate the adoption of learning strategies, have a positive effect on learners’ attention distribution and cognitive participation level (Artino, 2008; Jones et al., 2015). Therefore, the task value of the course can increase students’ self-regulated learning.

## General Discussion

Interactive Equivalence Theory can explain a clear majority of instructional interaction designs, as well as the inconsistent conclusions of previous studies to a certain extent. Despite few researchers pay attention to this theory, our findings verified the practicability of this theory, and the relationships between different types of instructional interactions and learning outcomes. Our study helps both scholars and practitioners understand the importance of different types of instructional interactions, and provide suggestions for optimizing resource allocation. In addition, since we confirmed the internal mechanisms between the instructional interactions and learning outcomes, instructional design can be adjusted purposefully and timely, so as to maximize the learning outcomes with limited resources and promote the further development of online education.

### The Impacts of Student–Student Interaction and Student–Content Interaction on Learning Satisfaction

The increase of SS can reduce the sense of isolation of learners in the online education environment. Information exchange between students and increased opportunities for exchange of knowledge and experience can help students understand the nature of the course and master the course content deeply. In turn, it can promote the improvement of academic performance and satisfaction (Kuo et al., 2013). SC is a process in which learners interpret, organize and reflect on new knowledge on the basis of integrating existing knowledge through internal dialogue (Moore, 1989). This process of intellectual interaction with content is a necessary process for education (Moore, 1989), which will lead to changes in learners’ understanding, perspective or thinking cognitive structure (Bernard et al., 2009). High quality course content and more SC can enable learners to have a more comprehensive understanding of the course, master the knowledge, and improve their course scores, learning satisfaction and task value perception.

Student–content interaction can predict learning satisfaction to a much greater extent than interpersonal interaction (SS and ST) in the statistical regression equation, which is consistent with the conclusion of previous studies that SC is the strongest predictor of learning outcomes (Kuo et al., 2013, 2014; Oyarzun et al., 2018). From the descriptive statistical results of the three instructional interactions, it can be inferred that students may pay too much attention to the learning content in the learning process and ignore or reduce interpersonal interaction (Li et al., 2014). In the light of the Interactive Equivalence Theory, when one of the three instructional interactions is at a high level, the impact of the other two instructional interactions on teaching effect and learning satisfaction will become less obvious (Anderson, 2003). There are differences in the importance that learners attach to the three instructional interactions (Rhode, 2009). SC is the easiest way for learners to operate and control, while ST is the hardest way to carry out in the online learning environment. The process of SC requires the least cognitive cost and technical restrictions (Anderson, 2003).

In addition, according to the observational learning in Social Learning Theory (Bandura, 1978), learners browse the interaction records between teachers and other students, as well as the interaction records between peers in the learning community, which can substitute and reinforce their own learning (Anderson, 2003). Interactive items between students and content (e.g., teacher notes, lecture slides) may serve as a substitute or complement to the teacher’s presence (Ke, 2013). With the continuous development of information technology (e.g., storage capacity), SS and ST will be likely to gradually transform into SC (Anderson, 2003). Therefore, in the regression of the three kinds of instructional interactions on learning satisfaction, the ST regression coefficient is not significant, and the SS regression coefficient is relatively small, which may be because the interaction between learners and content replaces the role of interpersonal interaction in e-learning to some extent.

### Task Value and Self-Regulated Learning as Mediator Variables

Interaction between classmates (Oyarzun et al., 2018) can facilitate student to evaluate course as useful, important and pleasure, that is, improving students’ perception of the task value of the course. Less interaction between learners in online education will make learners feel isolated and less interested in learning; The content quality of courses are main concerned aspects for students, and the interaction with content is the most critical instructional interactions for students in online education (Tsang, 2010; Rodriguez and Armellini, 2013). Hence, SS, SC will enhance students’ perception of the practicality and importance of learning courses, thus enhancing their learning satisfaction.

According to the interactive determinism principle of the Social Learning Theory (Bandura, 1978), individual cognition, behavior and environment are interdependent. Self-regulated learning is not an absolute functional state. Individuals will adjust their cognition, motivation and behavior under the influence of the external environment, then managing their own learning activities (Zimmerman, 1989). ST and SS belong to interpersonal interactions in instructional interactions (Moore, 1989), mainly referring to peer collaborative learning and teacher feedback. SC refers to students’ self-interpretation and reflection on learning, regardless of whether others are present or not. Through the regression results of Study 1, we know that SC can replace SS and ST without affecting the learning outcomes. In short, SC is a key factor influencing students to manage their own learning activities. Moreover, learning satisfaction increases with the increase of self-regulated learning (Puzziferro, 2008; Xu et al., 2017). Therefore, our findings are consistent with previous studies, that is, SC can affect learning satisfaction through the mediating effect of self-regulating learning.

Instructional interactions affect the results of online learning by influencing learners’ motivation and beliefs, personal behaviors and other factors (Pekrun, 2006). Motivational beliefs mainly include task value, self-efficacy and other aspects (Zhong et al., 2010). Autonomous learning is a personal act. Learners’ motivational beliefs can promote self-regulation and self-management of learning. Therefore, SC can not only affect students’ learning satisfaction through the simple mediation of task value and self-regulating learning, but also affect students’ learning satisfaction through the chain mediation of task value → self-regulating learning.

### Limitations and Implications for Future Research

There are still some areas that need to be improved. First, there may be some individual differences in self-reported instructional interactions. Some researchers have shown that students’ perception of instructional interaction is not necessarily equivalent to the level of instructional interactions actually designed in the curriculum (Thurmond and Wambach, 2004). Therefore, future research can adopt a combination of subjective and objective methods to measure the level of instructional interactions. Taking background behavior data analysis as an example, it can not only scientifically measure the level of instructional interactions, but also further analyze the relationship between actual instructional interactions and students’ perceived ones.

Secondly, this study adopts quasi-experimental research, which cannot strictly control other variables in field teaching context, and the subject that can be designed is also limited. In addition, only a single course can be investigated, rather than the combination design of different types of instructional interactions. Therefore, future research can design multiple groups of participants through laboratory experiments and extend them to different curriculum areas (Bernard et al., 2009).

## Conclusion and Implications for Practice

The current study is aimed to discuss the following research questions: (1) What are the relationships between instructional interactions and learning outcomes? (2) Whether there is interaction equivalence among SS, ST, and SC? (3) Whether task value and self-regulated learning play mediating roles in the above relationships? Through two empirical studies, that is, quasi-experimental research and online survey, the following conclusions are finally drawn:

(1) Not all of instructional interactions can significantly predict learning satisfaction. ST cannot significantly predict learning satisfaction, while SS and SC can significantly positively predict learning satisfaction; SC was the strongest predictor of learning satisfaction. (2) When the total amount of instructional interaction is constant, the types or quantity of instructional interactions can be adjusted according to the needs, which can effectively improve the learning outcomes to some extent. (3) SS can affect learning satisfaction *via* task value; SC can not only directly affect learning satisfaction, but also affect learning satisfaction through the simple mediations of task value, self-regulated learning and the chain mediations of “task value → self-regulated learning.”

Our research conclusion can make some supplement to the traditional instructional interaction theory and provide guidance for online course design. First of all, accepted conclusion holds that three types of instructional interaction are irreplaceable (Moore, 1989), and the increase of each type of instructional interactions can improve learning effect. This study points out that when content is properly designed, SC may replace interpersonal interaction; adjusting the type and number of instructional interactions as needed can effectively improve learning outcomes. Actually, since teaching resources are limited, it is unrealistic to improve learning effect by increasing the instructional interactions continuously. Teachers should selectively improve certain types of instructional interaction according to the needs of students; What students don’t need can be kept at a lower level or even eliminated. Besides, special attention should be paid to the quality of course content, and online platforms should minimize the technical difficulties of interpersonal interaction.

Secondly, the traditional instructional interaction study only considers the direct relationship without paying attention to the internal mechanism. Our study indicates that there are indeed indirect factors influencing the relationships between instructional interactions and learning outcomes, that is, task values and self-regulated learning. Therefore, teachers can design instructional interaction based on improving students’ perception of task value, and embed clear learning feedback cases in learning content to build students’ self-regulated learning.

## Data Availability Statement

The raw data supporting the conclusions of this article will be made available by the authors, without undue reservation.

## Ethics Statement

The studies involving human participants were reviewed and approved by the Ethic Institutional Review Board of Central China Normal University. The patients/participants provided their written informed consent to participate in this study.

## Author Contributions

All the authors contributed to the study design and approved the final version of the manuscript for submission. YT and FZ collected the data. XL and XYL analyzed the data, drafted and revised the manuscript.

## Conflict of Interest

The authors declare that the research was conducted in the absence of any commercial or financial relationships that could be construed as a potential conflict of interest.

## Publisher’s Note

All claims expressed in this article are solely those of the authors and do not necessarily represent those of their affiliated organizations, or those of the publisher, the editors and the reviewers. Any product that may be evaluated in this article, or claim that may be made by its manufacturer, is not guaranteed or endorsed by the publisher.

## References

[B1] Amer M. (2020). The impact of distance education on learning outcome in computer skills course in prince Sattam bin Abdulaziz university: an experimental study. *J. Curric. Teach.* 9 1–9. 10.5430/jct.v9n4p1

[B2] Anderson T. (2003). Getting the mix right again: an updated and theoretical rationale for interaction. *Int. Rev. Res. Open Distance Learn.* 4 126–141. 10.19173/irrodl.v4i2.149

[B3] Angelova B. Zekiri J. (2011). Measuring customer satisfaction with service quality using American customer satisfaction model (ACSI model). *Int. J. Acad. Res. Bus. Soc. Sci.* 1 232–258. 10.6007/ijarbss.v1i2.35

[B4] Artino A. R. (2008). Motivational beliefs and perceptions of instructional quality: predicting satisfaction with online training. *J. Comput. Assist. Learn.* 24 260–270. 10.1111/j.1365-2729.2007.00258.x

[B5] Bandura A. (1978). Social learning theory of aggression. *J. Commun.* 28 12–29. 10.1111/j.1460-2466.1978.tb01621.x 690254

[B6] Beldarrain Y. (2008). *Engaging the 21 st Century Learner: An Exploratory Study of the Relationship between Interaction and Achievement in the Virtual High School* Doctoral dissertation. Minneapolis, MN: Capella University.

[B7] Bernard R. M. Abrami P. C. Borokhovski E. Wade C. A. Tamim R. M. Surkes M. A. (2009). A meta-analysis of three types of interaction treatments in distance education. *Rev. Educ. Res.* 79 1243–1289. 10.1186/s12909-020-02483-w 33441140 PMC7805166

[B8] Bray E. Aoki K. Dlugosh L. (2008). Predictors of learning satisfaction in Japanese online distance learners. *Int. Rev. Res. Open Distrib. Learn.* 9 1–24. 10.19173/irrodl.v9i3.525

[B9] China Internet Network Information Center (CNNIC) (2020). *The 46th China Statistical Report on Internet Development.* Available online at: http://www.cnnic.cn/hlwfzyj/hlwxzbg/hlwtjbg/202009/t20200929_71257.htm (accessed June 6, 2021)

[B10] Dai Y. (2013). The dilemma and outlet of self-regulating learning in distance education: reflections based on the information interaction mode of distance education. *Mod. Distance Educ.* 2, 33–38.

[B11] Devkota K. R. (2021). Inequalities reinforced through online and distance education in the age of covid-19: the case of higher education in nepal. *Int. Rev. Educ.* 67 1–21. 10.1007/s11159-021-09886-x 33678863 PMC7925807

[B12] Ding X. Wu L. (2005). Exploration and reflection of SPOC: based teaching model in flipped classroom. *Distance Educ. China.* 3, 14–18+78.

[B13] Dong C. Cao S. Li H. (2020). Young children’s online learning during COVID-19 pandemic: Chinese parents’ beliefs and attitudes. *Child. Youth Serv. Rev.* 118:105440. 10.1016/j.childyouth.2020.105440 32921857 PMC7476883

[B14] Duan W. Zhang Y. (2010). The influence of academic goals orientationon academic achievement of tuition-free normal college students: self-regulated learning as a mediator. *Contemp. Teach. Educ.* 3 34–39.

[B15] Eccles J. S. Harold R. D. (1991). Gender differences in sport involvement: applying the eccles’ expectancy-value model. *J. Appl. Sport Psychol.* 3 7–35. 10.1080/10413209108406432

[B16] Faize F. A. Nawaz M. (2020). Evaluation and Improvement of students’ satisfaction in online learning during COVID-19. *Open Praxis* 12 495–507. 10.5944/openpraxis.12.4.1153

[B17] Field A. (2000). *Discovering Statistics using SPSS*, 3rd Edn. London: Sage.

[B18] Freeze R. D. Alshare K. A. Lane P. L. Wen H. J. (2010). Is success model in e-learning context based on students’ perceptions. *J. Inf. Syst. Educ.* 21 173–184.

[B19] Gong S. Han Y. Wang L. Gao L. Xiong J. (2016). The relationships among task value, academic emotions and online learning satisfaction. *E-Educ. Res.* 37 72–77.

[B20] Hayes A. F. (2009). Beyond baron and Kenny: statistical mediation analysis in the new millennium. *Commun. Monogr*. 76 408–420. 10.1080/03637750903310360

[B21] Jiang H. Islam A. A. Gu X. Spector J. M. (2021). Online learning satisfaction in higher education during the COVID-19 pandemic: a regional comparison between Eastern and Western Chinese universities. *Educ. Inf. Technol.* 26 1–23. 10.1007/s10639-021-10519-x 33814959 PMC8010491

[B22] Johnson M. L. Wards O. V. Dai T. (eds) (2014). Growth trajectories of task value and self-efficacy across an academic semester. *Univ. J. Educ. Res.* 2 10–18. 10.13189/ujer.2014.020102

[B23] Joksimovic S. Gasevic D. Loughin T. M. Kovanovic V. Hatala M. (2015). Learning at distance: effects of interaction traces on academic achievement. *Comput. Educ.* 87 204–217. 10.1016/j.compedu.2015.07.002

[B24] Jones S. H. Johnson M. L. Campbell B. D. (2015). Hot factors for a cold topic: examining the role of task-value, attention allocation, and engagement on conceptual change. *Contemp. Educ. Psychol.* 42 62–70. 10.1016/j.cedpsych.2015.04.004

[B25] Ke F. (2013). Online interaction arrangements on quality of online interactions performed by diverse learners across disciplines. *Internet High. Educ.* 16 14–22. 10.1016/j.iheduc.2012.07.003

[B26] Krchner H. Schne C. Schwinger M. (2021). Beyond level of self-esteem: exploring the interplay of level, stability, and contingency of self-esteem, mediating factors, and academic achievement. *Soc. Psychol. Educ.* 24 319–341. 10.1007/s11218-021-09610-5

[B27] Kuo Y. C. Walker A. E. Schroder K. E. E. Belland B. R. (2014). Interaction, internet self-efficacy, and self-regulated learning as predictors of student satisfaction in online education courses. *Internet High. Educ.* 20 35–50. 10.1016/j.iheduc.2013.10.001

[B28] Kuo Y. Walker A. E. Belland B. R. Schroder K. E. E. (2013). A predictive study of student satisfaction in online education programs. *Int. Rev. Res. Open Distance Learn.* 14 16–39. 10.19173/irrodl.v14i1.1338

[B29] Lei Y. Zhou Z. Tian Y. (2017). The impact of motivational belief on student engagement during online learning. *China Electron. Educ.* 2, 82–88.

[B30] Li Q. Wang Z. Chen L. (2014). Key points of instructional videos design in xMOOCs: video analysis based on cases. *J. Distance Educ.* 32 95–102.

[B31] Mary T. Xiao J. (2014). Online interaction: why it matters to use forums strategically. *Distance Educ. China.* 7, 15–23.

[B32] Mbydzenyuy N. E. Silungwe D. (2020). Teaching and learning in resource-limited settings in the face of the covid-19 pandemic. *J. Educ. Technol. Online Learn.* 3 211–223.

[B33] Miyazoe T. Anderson T. (2012). Interaction equivalency theorem: the 64-interaction design model and its significance to online teaching. *Aaou* 2012 1–17. 10.5204/ssj.v10i1.424

[B34] Moore M. G. (1989). Three types of interaction. *Am. J. Distance Educ.* 3 1–7. 10.1080/08923648909526659

[B35] Nabizadeh S. Hajian S. Sheikhan Z. Rafiei F. (2019). Prediction of academic achievement based on learning strategies and outcome expectations among medical students. *BMC Med. Educ.* 19:99. 10.1186/s12909-019-1527-9 30953500 PMC6451267

[B36] Nikou S. Maslov I. (2021). An analysis of students’ perspectives on e-learning participation–the case of COVID-19 pandemic. *Int. J. Inf. Learn. Technol.* 38 299–315. 10.1108/ijilt-12-2020-0220

[B37] Oyarzun B. Stefaniak J. Bol L. Morrison G. R. (2018). Effects of learner-to-learner interactions on social presence, achievement and satisfaction. *J. Comput. High. Educ.* 30 154–175. 10.1007/s12528-017-9157-x

[B38] Pekrun R. (2006). The control-value theory of achievement emotions: assumptions, corollaries, and implications for educational research and practice. *Educ. Psychol. Rev.* 18 315–341. 10.3109/0142159X.2012.643265 22364457

[B39] Puzziferro M. (2008). Online technologies self-efficacy and self-regulated learning as predictors of final grade and satisfaction in college-level online courses. *Int. J. Phytoremediation* 21 72–89. 10.1080/08923640802039024

[B40] Qi Q. Fang P. (2005). Task value studies in retrospect and prospec. *J. Psychol. Sci.* 2005 488–490.

[B41] Ray A. Bala P. K. Chakraborty S. Dasgupta S. A. (2021). Exploring the impact of different factors on brand equity and intention to take up online courses from e-Learning platforms. *J. Retail. Consum. Serv.* 59:102351. 10.1016/j.jretconser.2020.102351

[B42] Rhode J. F. (2009). Interaction equivalency in self-paced online learning environments: an exploration of learner preferences. *Int. Rev. Res. Open Distance Learn.* 10 239–287. 10.19173/irrodl.v10i1.603

[B43] Rodriguez B. C. P. Armellini A. (2013). Interaction and effectiveness of corporate e-learning programmes. *Hum. Resour. Dev. Int.* 16 480–489. 10.1080/13678868.2013.803753

[B44] Sher A. (2009). Assessing the relationship of student-instructor and student-student interaction to student learning and satisfaction in web-based online learning environment. *J. Interact. Online Learn.* 8 102–120.

[B45] Sucipto T. Efendi A. Hanif H. N. Budiyanto C. (2017). The influence of learning management technology to student’s learning outcome. *Online Submission* 1 11–18. 10.20961/ijpte.v1i1.4606

[B46] Sun N. Li H. (2011). Does perceived interaction necessarily generate customer satisfaction? The empirical research on the mechanism of experiential value, customer involvement and shopping task style. *Econ. Manage.* 33 1–38.

[B47] Thorpe M. Godwin S. (2006). Interaction and e-learning: the student experience. *Stud. Contin. Educ.* 28 203–221. 10.1080/01580370600947330

[B48] Thurmond V. Wambach K. (2004). Understanding interactions in distance education: a review of the literature. *Int. J. Instr. Technol. Distance Learn.* 1 9–25.

[B49] Tsang E. (2010). *Learner-Content Interactions and Learning Effectiveness: A Study of Student Perceptions.* Minneapolis, MN: Capella University.

[B50] Velle L. L. Newman S. Montgomery C. Hyatt D. (2020). Initial teacher education in England and the covid-19 pandemic: challenges and opportunities. *J. Educ. Teach.* 46 596–608. 10.1080/02607476.2020.1803051

[B51] Wan L. Du J. Jiang L. (2017). The quality management of open educational resources: research progress and enlightenment. *China Educ. Technol.* 2 55–63.

[B52] Wang J. Zhang W. Zhu Z. Zhen S. Mai Y. Li D. (2010). A modulating model for the impacting factors in self-regulated learning of college students. *Acta Psychol. Sin.* 42 262–270. 10.3724/SP.J.1041.2010.00262

[B53] Wang Z. (2016). Further analysis of the essence and concepts of instructional interaction in online distance education. *e-Educ. Res.* 37 36–41.

[B54] Xiao J. (2017). Learner-content interaction in distance education: the weakest link in interaction research. *Distance Educ.* 38 123–135. 10.1080/01587919.2017.1298982

[B55] Xie X. Zhang Y. (2004). Concept and origin of distant education. *Mod. Educ. Technol.* 14 25–28.

[B56] Xu X. Zhao W. Liu H. (2017). Factors influencing college students’ satisfaction in online learning. *Distance Educ. China* 508 43–50.

[B57] Yang S. Mo D. (2010). A case study on the teaching existence of online education course forum. *China Educ. Technol.* 39–44. 10.1016/j.heliyon.2020.e05733 33426320 PMC7775862

[B58] Yang W. C. Xiong C. P. Ding J. H. Jiang Y. C. (2014). Study on the influence elements and mechanism s of education information resources – structural equation model analysis on 296 questionnaires from middle school teacher. *China Electro Chem. Educ.* 5, 104–121.

[B59] Zeng J. Lu X. Yang Y. Wu X. Zheng Q. (2016). Research on influencing factors of distance learner satisfaction based on structural equation. *Distance Educ. China.* 8, 59–65+80.

[B60] Zhang Y. (2011). Classroom teaching evaluation to promote student development. *Sci. Technol. Inf.* 23:685.

[B61] Zhong D. Chen Y. Zhou H. (2010). The influence of motivational beliefs on self-regulation learning in Middle school students. *Chin. J. Clin. Psychol.* 18 657–659.

[B62] Zhou H. Long L. (2004). Statistical remedies for common method biases. *Adv. Psychol. Sci.* 12 942–950.

[B63] Zhu Z. Wang J. Zhang W. Ye Q. (2005). Constraction of self-regulated learning scale for college students. *Psychol. Dev. Educ.* 21 60–65.

[B64] Zimmerman B. J. (1989). A social cognitive view of self-regulated academic learning. *J. Educ. Psychol.* 81 329–339. 10.1037/0022-0663.81.3.329

